# Undifferentiated Spindle-Cell Carcinoma of the Gallbladder: A Report of a Case, an Immunohistochemistry Profile, and a Review of the Literature

**DOI:** 10.1155/2013/267194

**Published:** 2013-02-13

**Authors:** Kabir Bolarinwa Badmos, Laila Salah Seada, Fawaz Fahad Al Rashid, Hanan Abdulhafez Oreiby

**Affiliations:** ^1^Department of Anatomic and Molecular Pathology, College of Medicine, University of Lagos, P.O. Box 12003, Lagos 100254, Nigeria; ^2^Histopathology Unit, Laboratory Department, King Khalid Hospital, King Abdulaziz Road, P.O. Box 2049, Hail 81461, Saudi Arabia; ^3^Surgery Department, King Khalid Hospital, King Abdulaziz Road, P.O. Box 2049, Hail 81461, Saudi Arabia

## Abstract

Undifferentiated spindle-cell carcinoma is a rare gallbladder cancer with a worse or similar prognosis to the generally dismal outcome seen in most gallbladder cancer patients. We reported a case of SpCC, stage IV disease that was initially diagnosed as undifferentiated pleomorphic sarcoma, but deeper sections revealed few clusters of epithelioid clear cells. Although the tumour showed biphasic appearances on haematoxylin and eosin, it exhibited poor protein expression with most sarcoma markers being negative except for focal vimentin positivity. The CEA and CK7 were positive only in the epithelioid clear cell clusters while CD 68 positive was also focally positive in the spindle-cell component. The poor tumour differentiation coupled with advanced stage at presentation was partly responsible for the disease progression and patients' death one year after surgery. Early diagnosis and surgical intervention with better understanding of this tumour biology may offer improved prognosis and survival in this rare cancer.

## 1. Introduction

Undifferentiated spindle-cell carcinoma (SpCC) of the gallbladder is a rare gallbladder cancer comprising of predominantly sarcomatous elements admixed with carcinomatous elements [1]. The body of evidence from various reports suggest epithelial origin with sarcomatous dedifferentiation or stroma induction supported by focal immunopositivity of the mesenchymal component for epithelial markers [2, 3]. Morphological demonstration of biphasic appearances is essential for a diagnosis of spindle-cell carcinoma; however, in some instances, this may be difficult even after multiple sections and immunohistochemistry [4, 5]. 

The clinical features, pathogenesis, and prognostic determinants of SpCC are not fully understood probably due to the small number of individual case reports in most institutions. However, most reports tend to suggest a worse prognosis and poor survival compared to conventional gallbladder carcinoma following treatment [2, 5]. We herein present a case of undifferentiated gallbladder carcinoma, spindle-cell variant with liver and omental metastasis, the diagnostic challenges, immunohistochemistry, and a review of the literature.

## 2. Case Report

**Figure 1:**
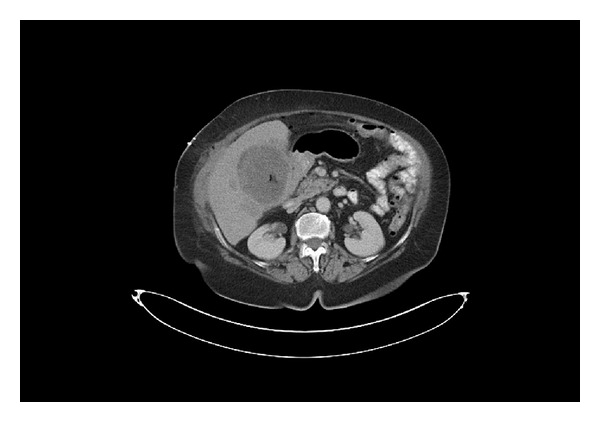
Computed tomography scan showing a 8 × 5 cm mass at the gallbladder bed (arrow) with two satellite lesions in the right hepatic lobe.

A 65-year-old diabetic woman presented with one-week history of abdominal pain and repeated vomiting. She admitted to a history of long standing recurrent abdominal pain. Significant examination findings were in the abdomen with right upper quadrant pain and a palpable mass. The initial clinical diagnosis was acute on chronic cholelithiasis, and she was admitted for laparoscopic cholecystectomy. The abdominal ultrasonography showed heterogeneous gallbladder lesion with calcification. Computed tomography scan revealed heterogeneous mass (8 × 7.6 × 7.4 cm) at the gallbladder bed and two satellite lesions in the right hepatic lobe (Figure 1). There was no paraaortic lymph node. The chest X-ray and CT scan were normal. The clinical diagnosis was reviewed to gallbladder cancer with hepatic metastasis. Complete blood count was normal except for leukocytosis (WBC 11, 800/mm^3^). Serum alkaline phosphatase was elevated (ALP −166 u/L). At laparotomy, the gallbladder tumour was adherent to the liver and omentum. The gallbladder-omental mass was excised together with biopsy from the liver nodule. 

**Figure 2:**
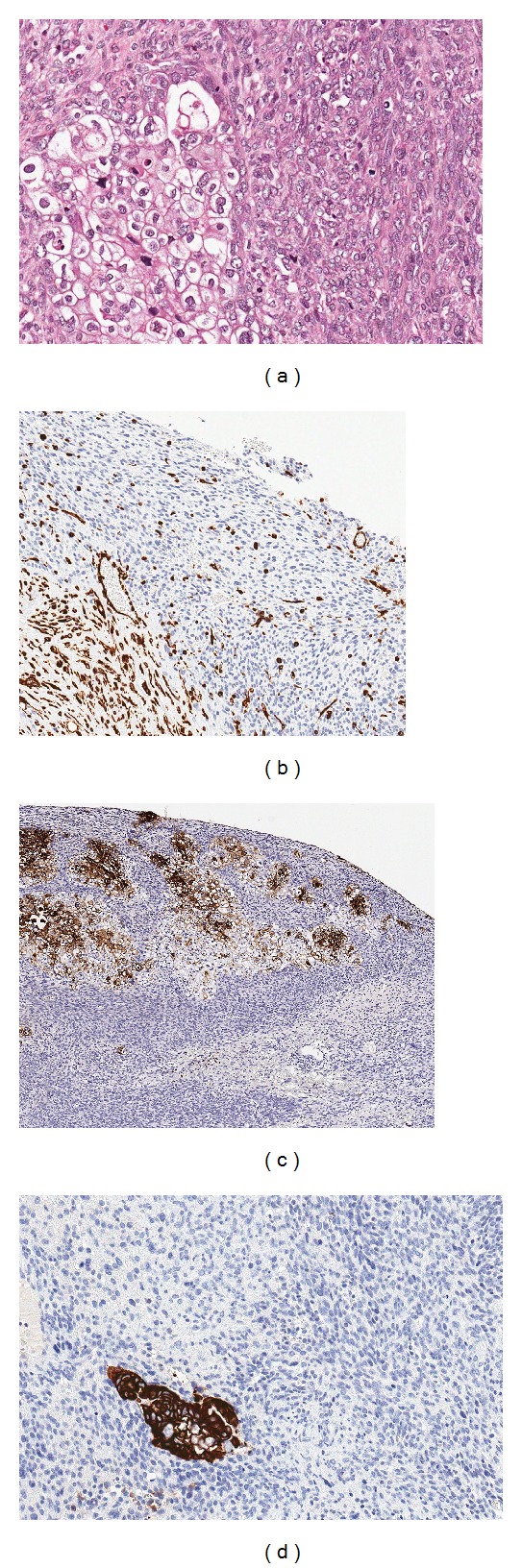
(a) Haematoxylin and eosin section showing malignant spindle cells with few clusters of malignant epithelioid cells. (b) Immunostaining showing focal positivity of the spindle cells for vimentin. (c) Immunostaining showing strong positivity of the epithelioid clear cells for CEA. (d) Immunostaining showing strong positivity of the epithelioid clear cells for CK7 at a focus.

The gallbladder mass with omental attachment was irregular, measured 8 × 5 × 4 cm with areas of haemorrhages. The liver biopsy measured 1 cm in diameter. Microscopic sections showed replacement of the gallbladder tissue as well as omental fat by malignant spindle-shaped tumour cells arranged in fascicles and storiform patterns with few clusters of malignant epithelioid cells (Figure 2(a)). Similar malignant spindle cells were seen infiltrating the liver tissues. Numerous tumour giant cells, mitoses of more than 5–7 per HPF, and necrosis were seen. There were numerous vascular channels and areas of haemorrhages. The initial histopathological diagnosis of undifferentiated pleomorphic sarcoma, stage IV (T3, Nx, M1) disease was made because the epithelioid clusters were only apparent after deeper sections were cut. Immunohistochemistry staining showed focal positivity for vimentin (Figure 2(b)) but negative for desmin, smooth muscle actin, S-100, CAM 5.2, EMA, and cytokeratin 20. The clusters of malignant epithelioid cells were positive for CEA and focally for CK7 (Figures 2(c) and 2(d)). Further immunostaining showed Hep Par 1 negativity and CD 68 focally positive in the spindle-cell components of the tumour. A final pathological diagnosis of undifferentiated spindle-cell carcinoma was concluded. The patient was referred for adjuvant radio-chemotherapy, but she demanded to be discharged against medical advice. The patient was lost to follow up and died 1 year after surgery of disease progression. 

## 3. Discussion

The majority of gallbladder cancer is conventional adenocarcinoma, whereas undifferentiated spindle-cell carcinoma (SpCC) is rare [6, 7]. The absence of glandular differentiation or areas reminiscent of epithelioid differentiation favours our initial assumption of undifferentiated pleomorphic sarcoma. However, serial sectioning of the tumour showed focal malignant epithelioid clusters with clear cytoplasm that were positive for CK7 and CEA embedded within the predominant spindle-cell tumour. It was this focal epithelioid differentiation that favoured our final diagnosis of SpCC even though the spindle-cell components were negative for all the epithelial markers and most of sarcoma markers except for focal immunopositivity of vimentin. According to the WHO classification of gastrointestinal tumours, four variants of undifferentiated carcinoma of the gallbladder are recognised out of which the spindle and giant cell type is the commonest [8]. The typical appearance is that of a sarcomatous lesion having variable admixture of spindle, giant, and polygonal cells with scattered multinucleated osteoclast-like giant cells as was seen in our case [4, 8].

In earlier reported cases, variable immunohistochemistry expressions have been noted. Kubota et al. demonstrated biphasic immunoreactivity to CK, EMA, and vimentin by the malignant epithelial and spindle-cell components [1]. Manouras et al. in their report showed that the tumour was immunonegative to most epithelial markers such as EMA, CEA, and AE1/AE3 but had focal positivity to CAM 5.2 and strong immunopositivity to mesenchymal markers (vimentin, SMA) [4]. The haematoxylin and eosin appearances of our case is that of a biphasic tumour with a predominant sarcomatous component focally positive for vimentin and few clusters of epithelial component with CEA and CK7 positivity. 

The varied protein expression coupled with rarity of this tumour contributes to its poorly understood pathogenesis. The admixture of the epithelial and mesenchymal tissues in undifferentiated carcinoma is probably due to dedifferentiation of epithelial cell into mesenchymal cells through metaplasia or by stromal induction. Sarcomatous change of carcinoma can be induced by radiotherapy, alterations to the *p53* gene and production of bone morphogenic proteins by cancer cells [9]. 

Despite improvement in radioimaging techniques, most cases of gallbladder cancers are diagnosed at advanced tumour stage with consequent dismal outcome following treatment. The key to improvement in the prognosis of gallbladder carcinoma would therefore be early detection prior to symptomatology by the patients. This would entail mass screening of population with high prevalence of gallbladder cancer using ultrasonography [10]. Regular screening with endoscopic ultrasonography in high-risk individuals such as those with congenital dilatation of common bile duct, abnormal pancreatobiliary ducts connections, reflux of pancreatic juice into the bile duct, or gallstones, as well as individuals with family history of gallbladder cancer, holds the key to early detection of gallbladder cancer [10]. 

The presence of serosa invasion and/or involvement of other organs as well as advanced stage were two identified factors that portend poor postsurgical outcomes in these patients. Our patient presented at advanced stage with liver and omental metastasis that probably resulted in her death due to progressive disease one year after surgery. The fact that curative surgery is near impossibility in most patients with gallbladder carcinoma due to advanced stage at presentation, patients and relatives should be thoroughly briefed about the biology of this tumour and prognosis following surgery. Newer chemo-radiotherapeutic agents as well as molecular targeted therapy would be essential to improved recurrence free survival following surgery. 

## References

[1] Kubota K, Kakuta Y, Kawamura S (2006). Undifferentiated spindle-cell carcinoma of the gallbladder: an immunohistochemical study. *Journal of Hepato-Biliary-Pancreatic Surgery*.

[2] Nishihara K, Tsuneyoshi M (1993). Undifferentiated spindle cell carcinoma of the gallbladder: a clinicopathologic, immunohistochemical, and flow cytometric study of 11 cases. *Human Pathology*.

[3] Diebold-Berger S, Vaiton JC, Pache JC, d’Amore ES (1995). Undifferentiated carcinoma of the gallbladder: report of a case with immunohistochemical findings. *Archives of Pathology and Laboratory Medicine*.

[4] Manouras A, Genetzakis M, Lagoudianakis EE (2009). Undifferentiated giant cell type carcinoma of the gallbladder with sarcomatoid dedifferentiation: a case report and review of the literature. *Journal of Medical Case Reports*.

[5] Guo KJ, Yamaguchi K, Enjoji M (1988). Undifferentiated carcinoma of the gallbladder. A clinicopathologic, histochemical, and immunohistochemical study of 21 patients with a poor prognosis. *Cancer*.

[6] Zhang L, Chen Z, Fukuma M, Lee LY, Wu M (2008). Prognostic significance of race and tumour size in carcinosarcoma of gallbladder: a meta-analysis of 68 cases. *International Journal of Clinical and Experimental Pathology*.

[7] Shaffer EA, Stinton LM (2012). Epidemiology of gallbladder disease: cholelithiasis and cancer. *Gut and Liver*.

[8] Hamilton SR, Aaltonen LA (2000). *World Health Organization Classification of Tumours. Pathology and Genetics of Tumours of the Digestive System*.

[9] Okabayashi T, Sun ZL, Montgomery RA, Hanazaki K (2009). Surgical outcome of carcinosarcoma of the gall bladder: a review. *World Journal of Gastroenterology*.

[10] Inui K, Yoshino J, Miyoshi H (2011). Diagnosis of gallbladder tumors. *Internal Medicine*.

